# Impact of Maternal HIV Seroconversion during Pregnancy on Early Mother to Child Transmission of HIV (MTCT) Measured at 4-8 Weeks Postpartum in South Africa 2011-2012: A National Population-Based Evaluation

**DOI:** 10.1371/journal.pone.0125525

**Published:** 2015-05-05

**Authors:** Thu-Ha Dinh, Kevin P. Delaney, Ameena Goga, Debra Jackson, Carl Lombard, Selamawit Woldesenbet, Mary Mogashoa, Yogan Pillay, Nathan Shaffer

**Affiliations:** 1 Centers for Disease Control and Prevention, Center for Global Health, Division of Global HIV/AIDS, Atlanta, Georgia, United States of America; 2 Centers for Disease Control and Prevention, National Center for HIV, Hepatitis, STD, and Tuberculosis Prevention, Division of HIV/AIDS Prevention, Atlanta, Georgia, United States of America; 3 Medical Research Council, Pretoria, South Africa; 4 Department of Paediatrics and Child Health, Kalafong Hospital, University of Pretoria, Pretoria, South Africa; 5 Medical Research Council, Cape Town, South Africa; 6 School of Public Health, University of the Western Cape, Bellville, South Africa; 7 US Centers for Disease Control and Prevention, Center for Global Health, Division of Global HIV/AIDS, Pretoria, South Africa; 8 National Department of Health, Pretoria, South Africa; 9 World Health Organization, Geneva, Switzerland; 10 United Nations Children’s Fund, New York, United States of America; University of Cape Town, SOUTH AFRICA

## Abstract

**Background:**

Mother-to-child transmission of HIV (MTCT) depends on the timing of HIV infection. We estimated HIV-seroconversion during pregnancy (HSP) after having a HIV-negative result antenatally, and its contribution to early MTCT in South Africa (SA).

**Methods and Findings:**

Between August 2011 and March 2012, we recruited a nationally representative sample of mother-infant pairs with infants aged 4-to-8 weeks from 578 health facilities. Data collection included mother interviews, child health-card reviews, and infant dried-blood-spots sample (iDBS). iDBS were tested for HIV antibodies and HIV-deoxyribonucleic-acid (HIV-DNA). HSP was defined as maternal self-report of an HIV-negative test during this pregnancy, no documented use of antiretroviral drugs and a matched HIV sero-positive iDBS. We used 20 imputations from a uniform distribution for time from reported antenatal HIV-negative result to delivery to estimate time of HSP. Early MTCT was defined based on detection of HIV-DNA in iDBS. Estimates were adjusted for clustering, nonresponse, and weighted by SA’s 2011 live-births.

**Results:**

Of 9802 mother-infant pairs, 2738 iDBS were HIV sero-positive, including 212 HSP, resulting in a nationally weighted estimate of 3.3% HSP (95% Confidence Interval: 2.8%-3.8%). Median time of HIV-seroconversion was 32.8weeks gestation;28.3% (19.7%- 36.9%) estimated to be >36 weeks. Early MTCT was 10.7% for HSP (6.2%-16.8%) vs. 2.2% (1.7%-2.8%) for mothers with known HIV-positive status. Although they represent 2.2% of all mothers and 6.7% of HIV-infected mothers, HSP accounted for 26% of early MTCT. Multivariable analysis indicated the highest risk for HSP was among women who knew the baby’s father was HIV-infected (adjusted-hazard ratio (aHR) 4.71; 1.49-14.99), or who had been screened for tuberculosis (aHR 1.82; 1.43-2.32).

**Conclusions:**

HSP risk is high and contributes significantly to early MTCT. Identification of HSP by repeat-testing at 32 weeks gestation, during labor, 6 weeks postpartum, in tuberculosis-exposed women, and in discordant couples might reduce MTCT.

## Introduction

Current guidelines in South Africa (SA) recommend testing pregnant women for human immunodeficiency virus (HIV) antibodies at the first antenatal care visit, retesting at 32 weeks gestation and again at labor, with the goal of this testing being the early identification of both existing undiagnosed HIV infection as well as incident infections occurring during pregnancy.[1] This testing intervention, combined with the 2013 World Health Organization (WHO) recommendations for early initiation of triple antiretroviral therapy (ART) as soon as HIV infection is identified in pregnant and breastfeeding women, is crucial to preventing mother to child transmission of HIV (PMTCT), preserving the mother’s health and reducing the number of HIV-infected children.[2] Even when a mother’s HIV infection is discovered late in pregnancy or at birth, the use of ART coupled with other interventions such as exclusive breastfeeding could result in substantial reductions in mother to child transmission (MTCT).[3, 4]

It has been hypothesized that the risk of MTCT may be higher for HIV-infected women who become infected during pregnancy than for those who are seropositive before pregnancy.[5] Levels of circulating HIV virus are known to be higher in the first few weeks of infection [6, 7], and typically remain high for the first 3–4 months after infection.[8, 9] Furthermore, the volume of maternal blood that a pregnant woman exposes her fetus to, through the placenta, increases throughout pregnancy. Thus the risk of MTCT could depend on the timing of HIV infection, the concentration of HIV in her blood and the amount of blood to which the fetus is exposed.[10] Few studies have been able to document such a relationship [10–12], and those that do [10–11] could not identify the time when incident infections occurred during pregnancy. Although several observational studies in Malawi and Uganda show that rates of incident HIV infection were higher in pregnant women than non-pregnant women, none of these studies identified risk factors for incident infection that could be used to develop practical public health interventions.[11–13]

We aimed to estimate population-based risks of HIV seroconversion of women during pregnancy after having a HIV-negative result antenatally and its contribution to early MTCT risk and HIV prevalence in infants aged 4–8 weeks at national level. Further, we estimated the timing of the seroconversions in order to inform recommendations for optimal retest time-points and identified risk factors to recommend potential interventions to reduce maternal incident infections during pregnancy.

## Methods

This study was nested within the evaluation of the effectiveness of the national PMTCT programs in SA (SAPMTCTE) which aimed to estimate MTCT risks and HIV prevalence in infants aged 4–8 weeks. Between August 2011 and March 2012, we conducted a facility-based survey designed to recruit a representative sample of infants aged 4–8 weeks from all nine provinces in SA.

### Study design

A total of 12200 participant samples were desired to measure the HIV prevalence among the 4–8 week infant population and MTCT risk measured at 4–8 weeks postpartum (early MTCT) at a confidence interval (CI) width of 1% to 2%, varied by province (S1 Table). Since annual coverage of the first Diphtheria/Tetanus/Pertussis vaccinations was >95% of live-births in SA, we estimated the number of HIV-exposed infants attending each facility for the first DPT vaccine doses, and the HIV prevalence among pregnant women attending the 1^st^ antenatal care (ANC) visit for the district where the facility was located. Our primary sampling unit, the health facility, was stratified into three strata providing (1) >300 infants receiving1^st^ DPT annually and located in a district with antenatal HIV prevalence ≥ 29% (the national ANC HIV prevalence), (2) >300 infants receiving the first DPT annually and located in a district with antenatal HIV prevalence <29%, and [3] 130–300 infants receiving the first DPT annually regardless of HIV prevalence. A total of 580 primary health facilities (ranging from 33 to 79 facilities per province) were randomly selected from 25 strata, representing each stratum and each of the nine provinces with probability proportional to size (PPS) method.[14] At the time of recruitment two selected study sites were undergoing renovations, and their clients were being referred to other facilities in the area which also had a possibility of being selected in the sample, so that data were collected from 578 total facilities. We determined the total number of infant dried-blood-spots (iDBS) samples for each selected facility proportionally to number of infants receiving the first DPT at the facility annually (S1 Table). Eligible caregiver/mother and infants pairs were consecutively selected from facilities from each stratum over three-weeks (four weeks in one province).

For each caregiver-infant pair, a trained nurse conducted a face-to-face interview with the caregiver using a standardized questionnaire, reviewed the child health card (CHC) and had an iDBS sample collected for HIV testing.

For this analysis, we only included mother-infant pairs in which the mother had ≥ 1 antenatal care (ANC) visit and their infants aged 4–8 weeks had provided an appropriate iDBS sample for HIV testing. We assessed current socio-demographic and socio-economic characteristics, and collected data on ANC, labor/delivery including gestational age at delivery, infant birth weight, all prior HIV testing history, PMTCT/ART interventions received, and partner’s HIV status. The mothers who reported being tested for HIV and receiving HIV-negative results during this pregnancy were asked the time (gestational age) of their last HIV test. We extracted data related to PMTCT interventions from each infant’s health chart.[15] Data were collected using pre-programmed handheld devices (cell-phones) and interview data were uploaded real-time into a web-based database.[16]

Pre-test counseling and parental consent was conducted and iDBS were obtained from infants from heel prick blood draw onto Munktell-TFN 5-spot paper. Infant test results were returned to participants using routine national systems; de-identified results were captured in the study database.

### Laboratory Testing

All iDBS samples were tested in the National Institute for Communicable Diseases, National Health Laboratory Services, Johannesburg, using standardized procedures. iDBS underwent serologic testing for HIV antibody using an enzyme immunoassay (EIA) [Genscreen HIV1/2 Ab EIA Version 2, Bio-Rad Laboratories, France]. All antibody-positive and 10% of negative specimens were re-tested using a second EIA [Vironostika HIV Uni-form II plus O, bioMḕrieux Clinical Diagnostics, Marcy-L’Etoile, France]. Specimens with discordant results were tested with and HIV-1Western blot [GS HIV-1, Bio-Rad France] and in all such cases (n = 4) the Western blot was negative and considered definitive.

iDBS specimens with concordant positive or discordant EIA results and from self-reporting HIV-positive mothers were tested using a qualitative deoxyribonucleic acid (DNA) Polymerase Chain Reaction (PCR) to determine infant’s HIV infection status [COBAS AmpliPrep/COBAS TaqMan—CAP/CTM—Qualitative assay version 1.0 assay, Roche Diagnostics, Branchburg, NJ]. Confirmed antibody-positive specimens indicated HIV-exposed infants (HEI). EIA and PCR-positive infants were defined as having confirmed early HIV infection.

### Definitions of the mother’s HIV status during pregnancy

**Fig 1:**
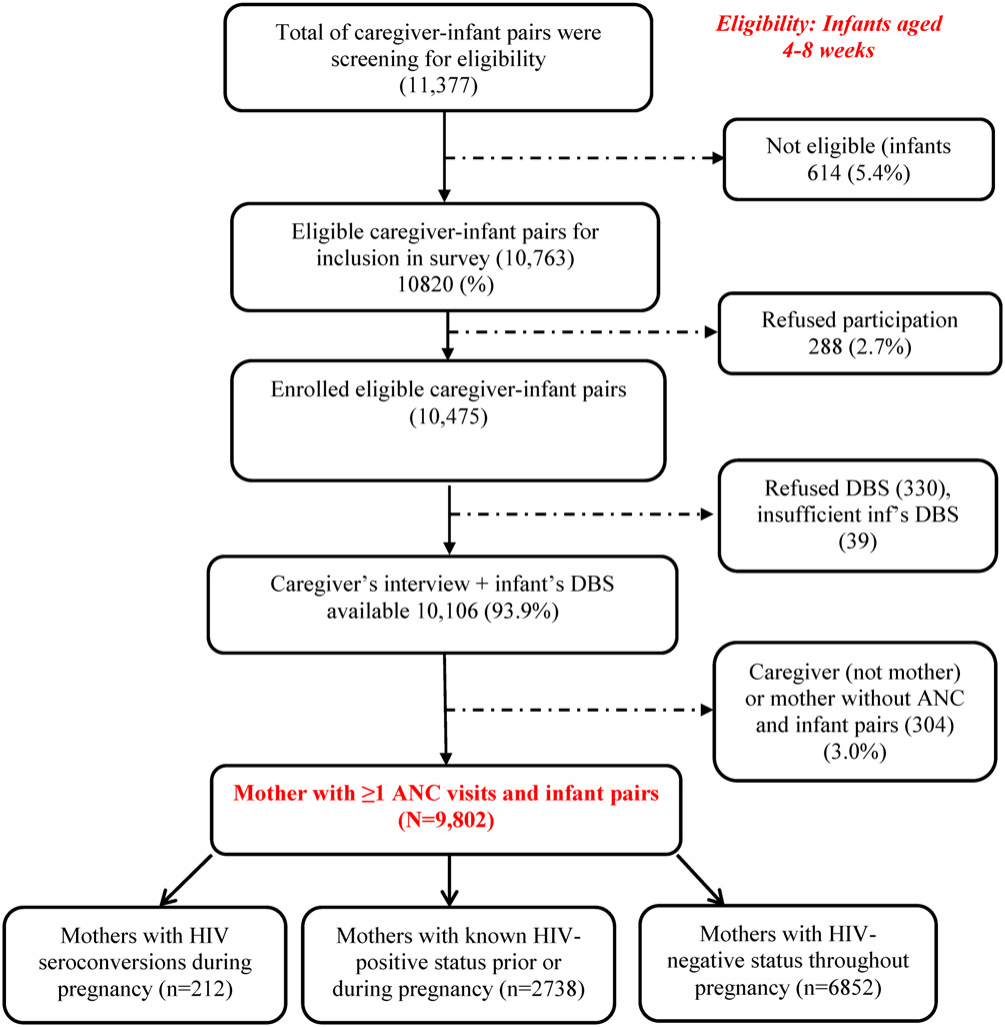
Profile of the evaluation of the effectiveness of the National Prevention of Mother-to-child transmission of HIV of the nested analysis of the maternal incident infection during pregnancy in South Africa, 2011–2012.

We used a composite of maternal self-report, the iDBS test result and information from the CHC to minimise bias in the classification of HIV status of mother’s due to self-report. In our sample 93% of mother-infant pairs had a CHC at interview; 61% of mother’s who self-reported as HIV-infected and 46% of those who reported their test result as HIV-negative had this documented on the CHC. Further 63% of mother’s who self-reported as HIV-infected had documentation of child prophylaxis on the CHC compared to less than 5% of those who reported being HIV-negative; mothers in this last group were considered to not have definitive information on HIV testing during ANC and were excluded from the seroconversion analysis (Fig 1). The remaining 9802 mother-infant pairs were categorized as follows:

1. Mothers with HIV-negative status throughout pregnancy: Mothers who met following four criteria (a) reported being tested for HIV, and (b) receiving HIV-negative result during this pregnancy, and (c) whose infant’s CHC did not record any HIV infection/exposure or any antiretroviral intake, and (d) whose iDBS collected at 4–8 weeks postpartum were negative for HIV antibodies (negative with HIV EIA test, or Western blot)
2. Mothers with known (prevalent) HIV infection during pregnancy: Mothers who reported having HIV-positive status diagnosed prior to this pregnancy or during pregnancy (at any ANC visits or at labor)
3. Mothers with seroconversion during pregnancy: Mothers who met following three criteria (a) reported being tested for HIV and having an HIV-negative result as the result of the latest test during this pregnancy, and (b) whose infant’s CHCs did not record any HIV infection /exposure or any antiretroviral intake, and (c) their iDBS collected at 4–8 weeks postpartum were positive for HIV antibodies (positive with two different HIV EIA tests, or Western blot)

### Statistical Methods

Data were analyzed as a retrospective cohort. Since HIV-positive pregnant women could only transmit HIV antibodies to their fetus through the placenta and the antibodies remain in HEI blood for an average of 13 weeks after delivery [17, 18], the mothers of infants with iDBS that were positive with HIV antibodies would have had positive HIV test results if they had been tested at delivery. We used these well-established data to impute seroconversion times in both descriptive analysis of the time to seroconversion as well as multivariate survival models that assessed the effect of potential risk factors on seroconversion as described below. We imputed time of seroconversion using a uniform distribution to assign an equal probability of seroconversion to each day between gestational age at delivery (the follow-up time-point to detect seroconversion) and the last reported negative HIV test during pregnancy (baseline time-point). To add variability to this imputation we repeated this procedure 20 times and calculated Kaplan-Meier cumulative incidence estimates using Taylor series linearization methods to calculate standard errors of the estimates to account for the survey design [19] and the methods of Rubin [20] to accounting for variance due to multiple imputation when estimating time of seroconversion. We did not directly ask about repeat testing during pregnancy, but estimated the proportion of mother’s who reported the latest negative HIV test obtained at or after 32 weeks gestation, as well as the proportion of mothers who reported that the latest negative test obtained after 32 weeks gestation was also more than 8 weeks after their first ANC visit, a potential marker for retesting according to the SA national PMTCT clinical guidelines.[1]

Multivariate analyses that again accounted for the survey sample design [19] in variance calculations were used to identify risk factors for HIV seroconversion included both a model for log odds of HSP, and a proportional hazards model for the time to seroconversion. Based on findings from previous studies, we considered a variety of possible confounders of the effects of covariates on HSP including characteristics of the mother (marital status, education, socio-economic status, age), pregnancy-related factors (gestational age at first antenatal visit, total number of ANC visits, testing for syphilis or whether the mother was screened for tuberculosis during this pregnancy, infant’s birth-weight, parity and number of live-children, where the baby was delivered, and who attended the birth) and partner’s HIV status in this analysis. The socio-economic status variable (SES) was a composite variable constructed using a clustering algorithm that considered 10 interview items (see S2 Table for a detailed description of the construction of this variable). Mother’s age, the number of ANC visits, total numbers of pregnancies and live-births, and infant birth-weight were included in bivariate and multivariate analyses as continuous variables; all other variables were categorical. Mother’s current marital status, SES, number of live-births, maternal HIV knowledge, where the baby was delivered and infant’s birth-weight did not change the effect by >10% from the full model (see S3 Table for the full model) and were not included in the final/reduced model.

Survey analysis methods employing Taylor series linearization for variance estimation were used to account for the sampling design including clustering of responses within facilities, stratification (for each of nine provinces, the study population was divided into strata based on number of administrations of the first DPT at each facility, HIV prevalence among the 1^st^ ANC visit in the prevalence—see the sampling section for more details) and non-response [19]. We treated all selected facilities that did not achieve the desired sample size (study sample), and the two clinics undergoing renovation where no participants could be recruited as non-responses in our analysis. Since this study was designed as a survey to recruit a national representative sample of infants aged 4–8 weeks, and given 98% of live-infants received the first DPT,[21] we used the distribution of numbers of live-births across the nine provinces in SA in 2011 to weight our estimates to population-level.[22]

All statistical analyses were carried out using SAS [version 9.3, SAS Institute, Cary, NC] and/or SUDAAN [Version 11.0.0, RTI International, Research Triangle Park, NC].

The protocol was approved by the United States Centers for Disease Control and Prevention (CDC), and by the institutional review board of the SA Medical Research Council (MRC). All participating caregivers and mothers provided written informed consent for themselves and their children. All consent forms were recorded in real-time during the interview using our study mobile devices and signed hard-copy consent forms were kept at the SAMRC in Cape Town per the approved protocol. All HIV test results were returned to participating mothers/caregivers through the clinic nurses as part of the routine services.

## Results

Of 10763 eligible infants, we recruited a total of 10106 (93.9%) mothers/caregivers from 578 facilities who consented to participate in the SAPMTCTE and whose iDBS specimen was sufficient for HIV testing. There were no significant differences at p-value of 0.05 in the distribution of province, mothers’ age, educational-level, the infants’ age or gender between the eligible infant-caregiver pairs who either refused to participate in the interview (n = 288) or to provide iDBS (n = 330), or whose iDBS samples were not sufficient for HIV testing (n = 39) and those who consented to participate to both sections (interviews and iDBS) of the study (Fig 1). Further, we excluded 304 mother/infant pairs who were either accompanied by caregivers who were not mothers or where the mother did not receive any ANC visit during pregnancy or where the mother’s self-report of HIV-negative status was refuted by a record of HIV treatment on the CHC. As a result, a total of 9802 infants aged 4–8 weeks and their mothers who had ≥ 1 ANC visit were eligible to be included in this analysis (Fig 1). Comparing the 304 excluded participants (3%) with the final sample (Table 1), the majority of the exclusions were caregivers (297/304; 54% were the child’s grandparent) and they reported that the mothers were significantly (p<0.05) younger (median age 21years), and more likely to be single (86%) but were not different (p>0.05) in terms of the distribution of the SES composite variable or proportion reporting vaginal delivery.

**Table 1:** Socio-demographic characteristics of participating mothers and infants by maternal HIV status during pregnancy, South Africa, 2011–2012.

	Mothers with HIV seroconversion during pregnancy			Mothers with known (prevalent) HIV-positive status prior or during pregnancy *			Mothers with HIV negative status throughout pregnancy		
	N** (n***) 25061 (212)	% (95%CI)	Mean (SD)	N** (n***) 348635 (2738)	%(95%CI)	Mean (SD)	N** (n***) 782600 (6852)	%(95%CI)	Mean (SD)
Maternal mean age (mean, Standard Deviation (SD))			26.6 (0.41)			28.0 (0.13)			25.2 (0.09)
Married status									
Single	18,482 (149)	73.7 (66.4–80.2)		270,153(2053)	77.5 (74.9–79.9)		565,958 (4890)	72.3 (70.2–74.3)	
Married/cohabiting	6,445 (61)	25.7 (19.3–33.0)		76,322 (665)	21.9(19.5–24.4)		213,570 (1931)	27.3 (25.3–29.4)	
Widow/Divorced	134 (2)	0.5 (0.2–2.7)		2,160 (20)	0.6 (0.4–1.0)		3,073 (31)	0.4 (0.2–0.6)	
Education level									
None	307 (4)	1.2 (0.2–3.8)		4,879 (41)	1.4 (1.0–2.0)		11,606 (104)	1.5 (1.2–1.9)	
Grade 1–7	4,572 (46)	18.2 (12.8–24.8)		57,518 (481)	16.5 (14.6–18.5)		93,001 (858)	11.9 (10.8–13.1)	
Grade 8–12	19,396 (156)	77.4 (70.6–83.2)		273,464 (2120)	78.4 (76.2–80.5)		626,273 (5487)	80.0 (78.2–81.5)	
Grade 12+	786 (6)	3.1 (1.0–7.2)		12,572 (94)	3.6 (2.7–4.7)		52,569 (400)	6.7 (5.7–7.8)	
Social economic status (SES)									
Average	15,032 (136)	60.0 (51.3–68.2)		218,361 (1792)	62.6 (58.6–66.5)		511,451 (4693)	65.4 (62.6–68.0)	
Lower	8,229 (62)	32.8 (25.0–41.4)		107,549 (773)	30.8 (27.1–34.8)		218,559 (1748)	27.9 (25.8–30.1)	
Lowest	1,800 (14)	7.2 (3.5–12.7)		22,614 (172)	6.5 (5.0–8.3)		52,230 (408)	6.7 (5.2–8.5)	
Parity (mean, SD)			2.3 (0.09)			2.5 (0.03)			2.1 (0.02)
No of live-children (mean,(SD))			2.1 (0.08)			2.3 (0.02)			2.0 (0.02)
Number of antenatal visit (mean, (SD))			4.3 (0.11)			5.1 (0.07)			4.9 (0.04)
Gestational age of 1^st^ antennal care visit (ANC) (mean, (SD))			18.4 (0.47)			17.6 (0.15)			18.0 (0.11)
Gave Birth at home	2346 (20)	9.4 (5.3–15.0)		15544 (130)	4.5 (3.5–5.6)		33168 (299)	4.2 (3.7–4.9)	
Birth attended by a physician	5523 (46)	22.0 (15.5–29.8)		93938 (686)	26.9 (24.7–29.3)		202556 (1738)	25.9 (24.4–27.3)	
Syphilis screening	19259 (158)	76.9 (69.5–83.2)		272374 (2040)	78.1 (75.1–81.0)		577274 (4996)	73.8 (70.7–76.6)	
Tuberculosis screening	11725 (106)	46.8 (43.7–60.3)		173356 (1343)	49.7 (45.9–53.5)		251870 (2431)	32.2 (29.3–36.2)	
Know Partners HIV status	16539 (141)	66.0 (57.9–73.5)		208842 (1662)	59.9 (57.2–62.5)		406770 (3506)	52.0 (50.0–54.0)	
Partner HIV positive	364 (4)	2.2		104823 (807)	50.2		5234 (46)	1.3	
Know week of receiving test result	17938 (148)	71.5 (64.6–77.8)		ND	ND		558484 (4846)	71.4 (68.3–74.3)	
Week of receiving test result, (mean, (SD))			25.0 (0.77)			ND****			24.5 (0.29)
Tested >32 weeks	5454 (40)	21.8 (15.0–29.8)					177583 (1428)	22.7 (20.0–25.6)	
And > 8 weeks after first ANC	3971 (26)	15.8 (9.9–23.5)					128257 (1024)	16.4 (14.1–18.8)	
Infant’s birth weight (kg, mean, (SD))			3.0 (0.03)			2.9 (0.02)			3.0 (0.01)
Gestational age at birth (weeks, mean (SD))			38.5 (0.19)			38.4 (0.07)			38.6 (0.04)
Caesarean delivery (%)	3626 (33)	14.5 (9.4–20.9)		81097 (596)	23.3 (20.9–25.7)		161525 (1378)	20.6 (19.4–22.0)	

*20 mothers who reported taking or having antiviral-drugs documented in the child health card reported “unknown HIV status were considered as “HIV-infected at or before current antenatal visit);

N** = weighted number for the live-birth in South Africa in 2011

n*** = actual number of the study participants

ND**** = Not documented/collected

Our weighted findings show that 30.0% (95%CI 28.4%–32.0%) of mothers were HIV-positive when pregnant. Of the mothers who reported having at least one ANC visit, being tested for HIV, and having an HIV-negative status as the latest HIV test result during pregnancy, and whose infant CHCs did not record any HIV infection /exposure or any antiretroviral exposure, 3.3% (95%CI 2.8%–3.8%) seroconverted before giving birth to the infant.

Table 1 shows characteristics of the study population divided into three groups based on the HIV status of the mother using the case definitions described in the method section: mothers with HIV seroconversion during pregnancy; mothers with known HIV-positive status (prevalent cases); and mothers with HIV-negative status throughout pregnancy. All three groups were made up of mostly multiparous, single women, of average composite SES status, who completed education through Grade 8, had the same mean number of pregnancies and live-births, and whose infants had similar mean birth- weight. Although all women reported having similar mean gestational age at first ANC (approximately 18 weeks), mothers with HIV-negative status throughout pregnancy as well as those with HIV seroconversion reported that they received the results of their last HIV test at a mean gestational age of 24.5 weeks and 25 weeks, respectively. Compared with mothers with HIV-negative status throughout pregnancy, mothers with known HIV-positive status during pregnancy were older (mean age 28 vs. 25 years), had a similar number of ANC visits (5.1 vs. 4.9), and were more likely to be screened for tuberculosis during the pregnancy (49.7% vs. 32.2%). Similarly, mothers with HIV seroconversion during pregnancy were older (mean age 26.6 vs. 25 years), more likely to report being screened for tuberculosis during this pregnancy (46.8% vs. 32.2%) and less likely to have had a delivery attended by a physician (22% vs. 25.9%) compared to those mothers with HIV-negative status throughout pregnancy. However, mothers with HIV seroconversion had fewer ANC visits (4.3 visits) compared to either mothers who reported being HIV-positive (5.1 visits) or those mothers with HIV-negative status throughout pregnancy (4.9 visits). A low percentage of both mothers who did (21.8%) and did not (22.7%) seroconvert reported testing negative at or after 32 weeks gestation. Similar and even lower percentages (15.8% and 16.4%, respectively) reported an HIV-negative test that both occurred at or after 32 weeks and that was more than 8 weeks after their first ANC visit. The proportion of mothers who reported knowing the father’s HIV status was low across all three groups, lowest among mothers with HIV-negative status throughout pregnancy (52%) and highest in women with seroconversion (66%). Of those who reported knowing the partner’s HIV status, 50.2% of women with known HIV-positive status during pregnancy, 2.2% of women with seroconversion, and 1.3% of mothers who were HIV-negative throughout pregnancy reported the father of the child was HIV-infected.

**Fig 2:**
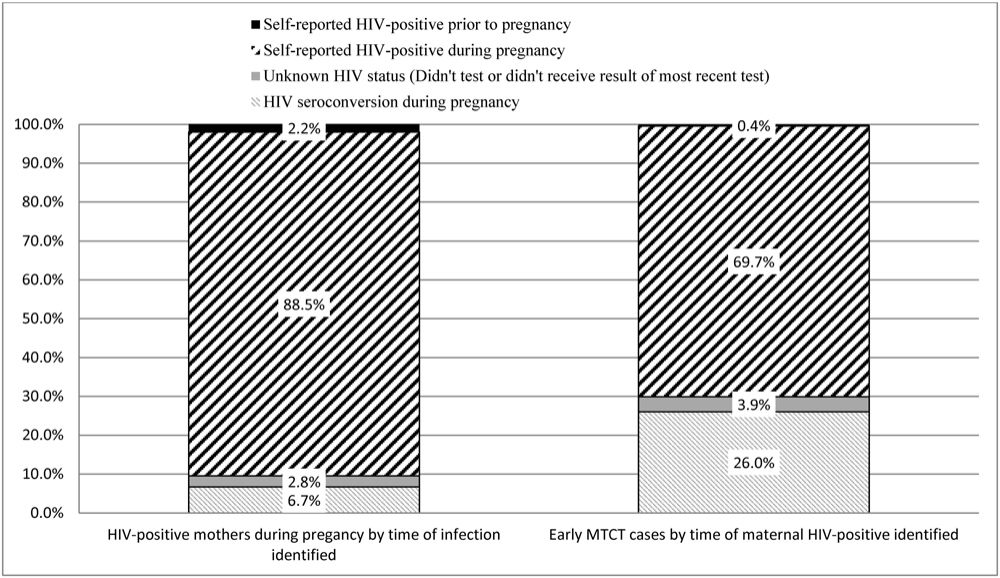
Weighted relative proportion of maternal HIV seroconversions during pregnancy and early mother to child transmission of HIV (MTCT) by time of maternal HIV infection identified group, South Africa, 2011–2012. This figure shows the distribution of awareness of HIV infection for all HIV-infected mothers, and for all mothers who transmitted HIV-infection to their infants. Mothers are categorized as a) knowing they were infected prior to this pregnancy (solid black) b) newly identified as HIV-infected during this pregnancy (upward diagonal lines) c) identified as HIV-infected by testing of infant dried blood spot specimens after reported not knowing their HIV status in our survey (solid grey box) and d) identified as HIV-infected by testing of iDBS after reporting testing HIV-negative in our survey, and with no other evidence of HIV infection reported on the infants child health card (light grey downward diagonal lines). Percentages are adjusted for the sampling design including clustering, design effects and nonresponse, and weighted to the population distribution of live-births for South Africa (SA) in 2011. It shows that, although women who seroconverted after a negative HIV test represent only 6.7% of the population of HIV-infected pregnant women in SA in 2011, these mothers accounted for 26.0% of all maternal to child transmission of HIV.

Although mothers with HIV seroconversion during pregnancy represent 6.7% (95%CI: 5.7%–7.8%) of all pregnant mothers infected with HIV (or HIV-exposed infants perinatally), these mothers account for 26.0% (95%CI: 17.1%–36.6%) of early MTCT cases in SA (Fig 2). Mothers who self-reported not testing for HIV or having unknown HIV status represent 2.8% (95%CI: 1.7%–4.3%) of the HIV prevalence (all mothers), and account for 3.9% (95%CI: 0.9%–10.3%) of early MTCT cases.

When comparing mothers who were HIV-negative throughout pregnancy and those with HIV seroconversion during pregnancy (Table 2), the highest risk for acquiring infection was for those mothers who reported knowing that the baby’s father was HIV infected (adjusted Hazard Ratio (aHR) 4.71; 95%CI 1.49–14.99). Having lower than average education (through grade 7 compared to those who had >12 years of education) (aHR 2.4; 95%CI 1.02–5.67), being screened for tuberculosis during pregnancy (aHR 1.82; 95%CI 1.43–2.32), and each year increase of maternal age (aHR 1.06; 95%CI 1.03–1.08) also significantly increased the risk of incident infection. Each additional follow-up ANC visit (aHR 0.90; 95%CI 0.83–0.97) and having the birth attended by a nurse (0.55; 95%CI 0.31–0.96) or doctor (aHR 0.49; 95%CI 0.27–0.90) reduced the risk of incident infection during pregnancy.

**Table 2:** Weighted risk factors of the maternal HIV seroconversion during pregnancy, South Africa, 2011–2012 (Reduced multivariate model).

		*Adjusted* * *Odds Ratio Estimates*			*Adjusted* * *Hazard Ratio Estimates*		
*Effect*	*Weighted row % of HIV seroconversion (3*.*29%)*	*Point Estimate*	*95% Wald Confidence Limits*		*Point Estimate*	*95% Wald Confidence Limits*	
Each additional year in mother’s age		1.06	1.03	1.09	1.06	1.03	1.08
Each additional ANC visit (over 1)		0.89	0.77	1.03	0.90	0.83	0.97
Maternal education completed							
No Education	2.83	1.22	0.35	4.24	1.22	0.36	4.14
Grade 1–7	5.09	2.45	1.02	5.85	2.40	1.02	5.67
Grade 8–12	3.18	1.86	0.84	4.15	1.84	0.83	4.05
>12 years	1.57	Ref.			Ref.		
Baby’s father HIV status							
HIV-negative	2.18	Ref.			Ref.		
HIV-infected	10.74	5.03	1.45	17.41	4.71	1.49	14.99
Don’t know	4.31	1.91	1.42	2.55	1.88	1.41	2.51
Tuberculosis screening during pregnancy							
Yes	4.64	1.81	1.41	2.32	1.82	1.43	2.32
No		Ref			Ref.		
Birth attendant							
Traditional healer/home delivery	6.69	Ref.			Ref.		
Nurse/Midwife	3.33	0.52	0.29	0.94	0.55	0.31	0.96
Doctor	2.70	0.47	0.25	0.88	0.49	0.27	0.90

*Adjusted for all listed covariates, total pregnancies including the index pregnancy, and the amount of support the mother reported receiving during pregnancy weighted to account for sampling design and non-response, and with variance estimation via Taylor series linearization to account for sampling design (see methods). Other potential confounders of the effects listed in this table, including mother’s marital status, Socio-economic status, number of living children, maternal knowledge of modes of HIV transmission, whether the infant was born at home, in a hospital or clinic and birth weight of the baby in kilograms were assessed but did not change the reported effects by more than 10% from the full model (see S3 Table) and therefore are not included in the final model presented above.

**Fig 3:**
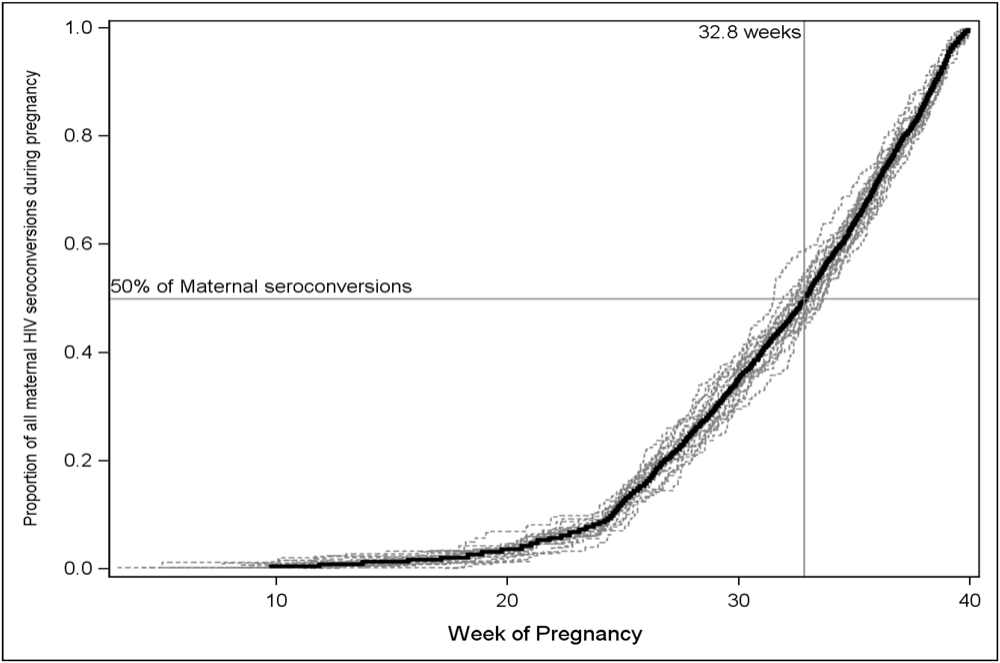
Estimated time of seroconversion for mothers with a negative HIV test result during PMTCT but became infected during their current pregnancy, South Africa, 2011–2012. This figure shows the results of a Kaplan-Meier (KM) time to event analysis of the time of seroconversion for 20 imputations of the weighted sample of 25,061 mothers represented by 211 sampled mothers who tested negative for HIV during this pregnancy and then became infected before they delivered their baby. Dashed lines represent the individual KM estimates for each of 20 imputations of the time of seroconversion, and the dark black line is the mean of these imputations. Median time of seroconversion was estimated to be 32.8 weeks of gestation, ranging from 31.5 to 33.6 across the imputations.

Based on reported gestational age at the last known negative HIV test result during pregnancy and at delivery, we estimated that the mean time of the HIV seroconversion during pregnancy was 31.8 weeks gestation (95%CI: 30.7–32.9). Additionally, we estimated that 28.3% (95%CI: 19.7–36.9) of women were infected after week 36 of gestation (Fig 3).

**Fig 4:**
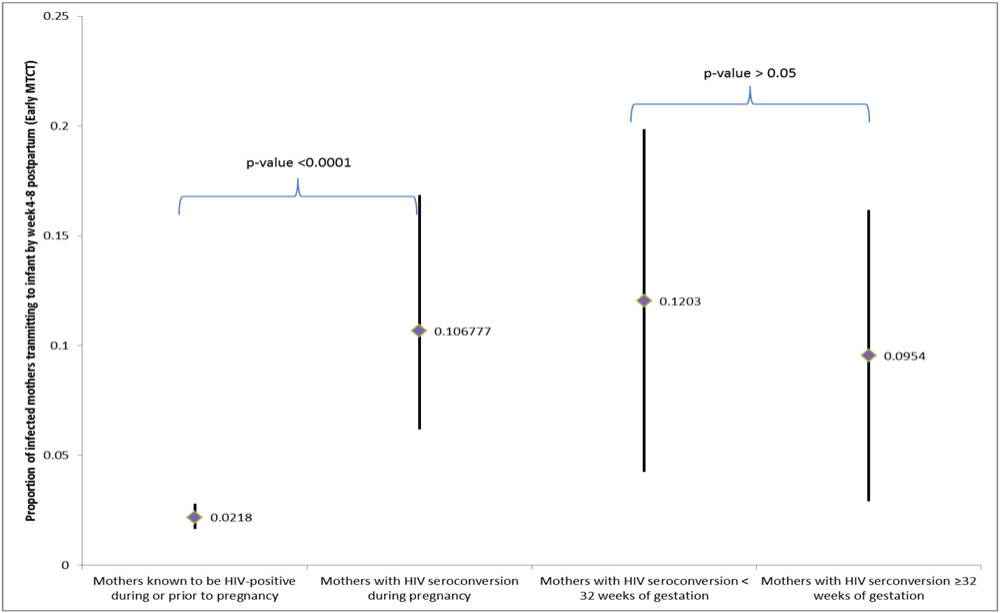
Weighted risk of early mother to child transmission among mothers with HIV seroconversions during pregnancy compared to mothers with reported HIV-positive status prior to the current pregnancy. This figure illustrates the estimate (diamonds) and 95% confidence intervals (vertical lines) for the proportion of HIV-infected mothers who had transmitted HIV to their infants by 4–8 weeks postpartum. Proportions are adjusted for the survey sampling design including clustering, design effects and nonresponse, and weighted to the population distribution of live-births for South Africa (SA) in 2011. The left panel of the graph shows a significantly higher proportion of mothers who seroconverted during pregnancy transmitted HIV to their infants than did mothers whose HIV infection was known to them before or during this pregnancy. The right panel subsets the women who seroconverted during pregnancy by the estimated week of gestation in which they seroconverted. Estimates of time of seroconversion were imputed using a uniform distribution to assign an equal probability of seroconversion to each day between delivery and the last reported negative HIV test during pregnancy. The graph shows results based on combined analysis of 20 imputations of this variable, and shows that the confidence intervals for the estimated proportion of mothers transmitting to their infants did not differ whether the mother was estimated to have seroconverted before or after 32 weeks gestation.

Fig 4 shows that early MTCT risk among mothers with HIV seroconversion during pregnancy was significantly higher than that observed among those mothers known to be HIV-positive during pregnancy (10.7% (95%CI: 6.2%–16.8%) vs. 2.2% (95%CI: 1.7%–2.8%), p<0.01). Considering the estimated time of HIV seroconversion, we found that early MTCT risk was similar between the group of mothers whose seroconversion occurred ≥ 32 weeks of gestation, (the recommended retest time point for those with an HIV-negative results in SA), and those mothers estimated to have acquired HIV infection before 32 weeks (12.0%vs. 9.5%, p>0.05).

## Discussion

Results from this study show that, in 2011–2012, an estimated 3.3% (n = 25 061) of mothers in SA who had at least one HIV-negative test result antenatally seroconverted during pregnancy. Compared with the national estimate of 1.5% HIV incident infection risk among women aged 15 to 49 years in SA (2005–2008)[23], and with the 2.8% risk estimated by Rehle group [24], our finding indicates that pregnant women have a higher risk of acquiring HIV in pregnancy. The finding supports Moodley et al [25, 26], who reported 3.0% risk of incident infection between the first ANC visit and 36–40 weeks of gestation in three provinces of SA. Similarly, Kinuthia et al [27] reported higher (2.6%) risk during pregnancy than during breastfeeding in Kenya. Gray et al [11] also reported a higher risk during pregnancy (2.1%) as compared with non-pregnant or breastfeeding women (1.2%) in Uganda.

We also found that less than 60% of the mothers knew the HIV status of the infant’s fathers during pregnancy, indicating very low rates of HIV disclosure among sexually active couples. Although only 2.2% of women with HIV seroconversion during pregnancy reported the infant’s father was HIV-positive, their risk of acquiring HIV increased by 4.7 fold (aHR = 4.71) compared with women with a reported HIV-negative partner. We believe that the actual risk of HIV acquisition due to discordance (HIV-negative pregnant women and HIV-positive male sex-partner) during pregnancy may be much higher than our measurement because many fathers whose female partners did not know their status may have not been diagnosed or disclosed HIV infection, which partially explains the elevated risk for HIV seroconversion (aHR = 1.88) within this group of mothers. These findings support the WHO recommendation of offering couples counselling and testing to all pregnant women at PMTCT settings.

Although data on sexual behaviors were not collected in our study, other investigators [11] reported that of 3134 pregnancies in Uganda, 84% reported being sexually active during pregnancy and only 1% of pregnant women reported using a condom consistently during pregnancy. Moodley et al [26] also reported that amongst HIV-seroconverted pregnant women only 3.0% reported not having sex and only 2.8% reported using a condom during pregnancy. These findings again suggest that women in discordant couples were at significantly higher risk of acquiring HIV during pregnancy. A multi-country clinical trial [28] has provided strong evidence for the WHO recommendation [2] that HIV-positive partners in sero-discordant relationships should take ARVs and use condoms to reduce the risk of HIV incident infections in their partners. Additionally, encouraging HIV-negative women to use condoms during the pregnancy and breastfeeding to prevent horizontal transmission has also been recommended as part of HIV post-test counselling guidance as an early and effective measure before their sexual partner’s HIV-status is confirmed. There are limited studies on accessibility and acceptability of condoms among pregnant women, and the public health feasibility of this approach. A study in the United States [29] showed that there was a significant increase in condom use in pregnant women at high risk for sexually transmitted disease (from 33.3% to 84.6%) when condoms were accessible and distributed free of charge, along with health education.

We found that mothers had an 81% increased risk of HIV seroconversion if they underwent tuberculosis screening. Under the guidelines at the time in SA (the 2010 PMTCT clinical guidelines) [1] these women were likely to have reported being exposed to tuberculosis or have tuberculosis symptoms, resulting in the offer of screening. We also found that a significantly higher proportion of mothers who reported having HIV-positive status reported being screened for tuberculosis during pregnancy compared with those mothers with reported HIV-negative status throughout the pregnancy (49.7% vs. 32.2%). Since we did not record tuberculosis cases identified through the screening as well as history of the tuberculosis exposure, we could not establish the causal pathway between tuberculosis and HIV seroconversion within this study. However, we believe provision of a package of both tuberculosis screening and HIV testing [30] would improve early detection of both HIV infections, including incident infection, and tuberculosis infection, especiallyin settings with high prevalence of both HIV and tuberculosis like SA.

Since we recruited infants aged 4 to 8 weeks from August 2011 to March 2012, our participating mothers first received ANC and PMTCT services provided within November 2010 to June 2011, under the 2010 national PMTCT guidelines.[1] This means that pregnant women should have been offered an HIV test at the first ANC visit and those who were HIV-negative should have been offered another HIV test at 32 weeks gestation per the guidelines. However, we found the mean of gestational age when these women received their last reported HIV-negative result was 25 weeks instead of 32 weeks gestation, and less than 17% reported a test at or after 32 weeks that was also more than 8 weeks after their first ANC visit. A report of the national PMTCT program found that 98% of ANC attendees were tested for HIV at the first ANC visit [21], but, many of these mothers are engaging ANC late and not enough of the mothers who test HIV-negative early in pregnancy are being retested. We estimate that more than 50% of the seroconversions we observed occurred after 32 weeks gestation; therefore, even if they had been retested according to the current re-testing guidelines, these women would not have been diagnosed with HIV before delivery. Since the risk of early MTCT was high and not statistically different between those women with seroconversion before or after 32 weeks of gestation, retesting not only at 32 weeks gestation, but also at 36 weeks and/or during labor could help identify these women in order to administer ART before birth, and thereby help to reduce the early MTCT in this group. Since ART can also reduce MTCT risk during breastfeeding, retesting during postpartum (i.e., at 6 weeks postpartum) could further reduce late MTCT.

Although maternal HIV seroconversions represented only 2.2% (n = 25 061) of all mothers giving birth, they accounted for 26% of early MTCT infections in infants (of 10 330 HIV-infected infants due to early MTCT, 2686 HIV-infected infants who acquired HIV infection perinatally were born to 25061 mothers who seroconverted during pregnancy). If the HIV-positive status of those seroconverted mothers were detected before giving birth, they could have received proper ART immediately during pregnancy, labor, and throughout breastfeeding to prevent MTCT and reduce the early MTCT risk from the current risk of 10.7% to as low as 2.2% (Fig 3). If HIV infection had been detected during pregnancy, the national PMTCT programs could have averted as much as 2135 additional HIV-infected infants aged 4–8 weeks, and more during breastfeeding.

Our findings have some limitations. We were not able to estimate risk of HIV seroconversion among those who acquired HIV after being pregnant but were diagnosed as HIV-positive at the first or subsequent ANC visit(s). Additionally, our estimates did not include HIV exposure or transmission among those infants whose mothers (3%—Fig 1) did not attend ANC nor those who did not bring their infants to public health facilities for the first DPT immunization (<5% of live-births). Our estimates were based on proportion of mothers who reported antenatal HIV-negative status verified by a lack of ARV and HEI documented in the infant’s routine CHCs, and whose infant’s DBS were positive with HIV antibodies indicating the mother seroconverted after the last known HIV-negative status. Only 46% of HIV-negative test results during ANC were both reported by the mother and verified on the CHC. As a result, we may overestimate the risk of HIV seroconversion if some women considered to have seroconverted had failed to disclose their HIV infection during the interview and also had errors in documentation of ARV use on the CHC. However, such mothers would still represent missed opportunities for HIV diagnosis during pregnancy and be at increased risk of transmission because they and their infant did not receive prophylaxis. Although we believe that the uniform distribution approach is the most appropriate and conservative method to estimate time to seroconversion, for example compared to an interval censored data approach analysis, we possibly biased our estimates of timing of seroconversion by giving too much weight to the possibility of infections acquired later in pregnancy if sex frequency were to go down toward the end of pregnancy. However, while this might increase the proportion of infections detectable at week 32 of gestation, it is not likely to eliminate the possibility of seroconversion after this time period. The timing of seroconversion may be biased because the women may misreport the gestational age when the last negative test result was obtained (baseline time point); however, we don’t think this factor would bias our seroconversion risks. Estimates of association for risk factors of maternal seroconversion could be affected by both recall and social-desirability biases resulting from maternal self-report. However, we are unable to predict either the magnitude or the direction of these biases.

This is the first nationally representative estimate of the risk of HIV seroconversion among women who had at least one HIV-negative test result during pregnancy in SA. The findings highlight a few emergent priorities for the national PMTCT program including: repeat testing at 32 and 36 weeks gestation, at labor, and 6 weeks postpartum; couples counseling and testing and early initiation of ART for HIV-positive partners in sero-discordant partnerships; promotion of using condoms during pregnancy; and provision of HIV testing and tuberculosis screening as a package in ANC settings. Successfully implementing these interventions could help in early detection of incident HIV infections occurring during pregnancy, and early initiation of ART to reduce MTCT risk during pregnancy and breastfeeding.

## Supporting Information

S1 Table Survey Sample Size Calculations. (DOCX)

S2 Table Distribution of individual variables that contributed to the overall socio-economic status (SES) score levels of average, lower than average and lowest SES. (DOCX)

S3 Table Weighted risk factors of the maternal HIV seroconversion during pregnancy, South Africa, 2011–2012 (Full model). (DOCX)
