# Correction: Impact of Maternal HIV Seroconversion during Pregnancy on Early Mother to Child Transmission of HIV (MTCT) Measured at 4-8 Weeks Postpartum in South Africa 2011-2012: A National Population-Based Evaluation

**DOI:** 10.1371/journal.pone.0130321

**Published:** 2015-06-04

**Authors:** Thu-Ha Dinh, Kevin P. Delaney, Ameena Goga, Debra Jackson, Carl Lombard, Selamawit Woldesenbet, Mary Mogashoa, Yogan Pillay, Nathan Shaffer

There are errors in the author affiliations. The affiliations should appear as shown here: Thu-Ha Dinh^1^, Kevin P. Delaney^2^, Ameena Goga^3,4^, Debra Jackson^5,6^, Carl Lombard^7^, Selamawit Woldesenbet^7^, Mary Mogashoa^8^, Yogan Pillay^9^, Nathan Shaffer^10^

**1** Centers for Disease Control and Prevention, Center for Global Health, Division of Global HIV/AIDS, Atlanta, Georgia, United States of America, **2** Centers for Disease Control and Prevention, National Center for HIV, Hepatitis, STD, and Tuberculosis Prevention, Division of HIV/AIDS Prevention, Atlanta, Georgia, United States of America, **3** Medical Research Council, Pretoria, South Africa, **4** Department of Paediatrics and Child Health, Kalafong Hospital, University of Pretoria, Pretoria, South Africa, **5** School of Public Health, University of the Western Cape, Bellville, South Africa, **6** United Nations Children's Fund, New York, United States of America, **7** Medical Research Council, Cape Town, South Africa, **8** US Centers for Disease Control and Prevention, Center for Global Health, Division of Global HIV/AIDS, Pretoria, South Africa, **9** National Department of Health, Pretoria, South Africa, **10** World Health Organization, Geneva, Switzerland.

The data sharing statement is incorrect. The correct statement is:

The data are bound by ethical and legal restrictions. To access the data of the South Africa's Prevention of Mother to Child Transmission Effective study, investigators that are not part of the study team should submit a concept proposal to Thu-Ha Dinh, MD, MS (US Centers for Disease Control and Prevention (CDC), Principal investigator), Ameena Goga, MD, MS (South Africa Medical Research Council (MRC), Principal Investigator) and Debra Jackson (University of the Western Cape/UNICEF, Principal Investigator) for approval. Investigators with an approved concept proposal must apply for guest researcher status obtain access to a workstation and the data. Additionally they will need to complete data security and confidentiality training, and to sign data use and nondisclosure agreements.
