# Supplementary material for: Impact of Maternal HIV Seroconversion during Pregnancy on Early Mother to Child Transmission of HIV (MTCT) Measured at 4-8 Weeks Postpartum in South Africa 2011-2012: A National Population-Based Evaluation
Source: PLoS One. 2015 May 5;10(5):e0125525. doi: 10.1371/journal.pone.0125525 (PMC4420458; doi:10.1371/journal.pone.0125525)
Supplement: S1 Table — (DOCX) [file pone.0125525.s001.docx]

**Table S1. Sample size calculation**

|  | **ANC HIV Prev. 2008** | **% ANC HIV test** | **% babies on PMTCT** | **Estimated Coverage (%tested X %admin to baby)** | **No PMTCT coverage** | **MTCT in exposed assuming sd NVP =15% & untreated=29% (Rollins)*** | **Overall Pop. Prev.** | **30% relative precision in each province** | | | | **Varying relative precision across provinces** | | | |
| --- | --- | --- | --- | --- | --- | --- | --- | --- | --- | --- | --- | --- | --- | --- | --- |
|  |  |  |  |  |  |  |  | **Error margin with 30% relative precision (RP)** | **RP** | **SS for 30% RP** | **Sample size for design effect (DE)** of 2 & RP 30%** | **Error margin with RP** | **Varying RP by province** | **Sample size using varying RP without DE** | **Sample size using varying RP with DE** of 2** |
| **ZA** | 29 | 67 | 47 | 31.5% |  | 24.6% | 7.1% | 2.1 | 30 | 575 | 1150 |  |  |  |  |
| **EC** | 24 | 73 | 35 | 25.6% | 74.5% | 25.4% | 6.1% | 1.8 | 30 | 680 | 1360 | 1.8 | 30% | 700 | 1400 |
| **FS** | 29 | 70 | 52 | 36.4% | 63.6% | 23.9% | 6.9% | 2.1 | 30 | 560 | 1120 | 2.0 | 29% | 617 | 1300 |
| **GP** | 31 | 65 | 27 | 17.6% | 82.5% | 26.5% | 8.2% | 2.5 | 30 | 463 | 926 | 2.0 | 24% | 723 | 1800 |
| **KZN*** | 37 | 66 | 52 | 34.3% | 65.7% | 21.4% | 7.9% | 2.4 | 30 | 485 | 970 | 2.0 | 25% | 699 | 1400 |
| **LP** | 20 | 74 | 54 | 40.0% | 60.0% | 23.4% | 4.7% | 1.4 | 30 | 878 | 1756 | 1.5 | 32% | 703 | 1400 |
| **MP** | 34 | 56 | 36 | 20.2% | 79.8% | 26.2% | 8.9% | 2.7 | 30 | 428 | 856 | 2.0 | 22% | 779 | 1600 |
| **NC** | 14 | 81 | 70 | 56.7% | 43.3% | 21.1% | 2.9% | 0.9 | 30 | 1336 | 2672 | 1.8 | 60% | 350 | 700 |
| **NW** | 29.9 | 86 | 50 | 43.0% | 57.0% | 23.0% | 6.9% | 2.1 | 30 | 560 | 1119 | 2.0 | 29% | 601 | 1200 |
| **WC*** | 15 | 97 | 75 | 72.8% | 27.3% | 13.0% | 1.9% | 0.6 | 30 | 1989 | 3978 | 1.0 | 51% | 716 | 1400 |
| **TOTAL** | | | | | | | | | | **7379** | **14758** |  |  |  | **12200** |

*EC – Eastern Cape Province; FS – Free State Province; GP = Gauteng Province; KZN = Kwa-Zulu Natal Province; LP – Limpopo Province; MP – Mpumalanga Province; NW – North West Province; NC – Northern Cape Province; WC – Western Cape Province. Prev. – prevalence ANC = antenatal clinic; RP = relative precision; sdNVP = single dose nevirapine; Pop. = population; RP = relative precision; DE = design effect*

*ANC HIV seroprevalence from the 2008 antenatal survey, published in 2009 and immunisation coverage data from the 2007 District Health Information system (DHIS)*

**WC and KZN assume full coverage dual therapy - Rollins KZN Study is 7%*

*** Design Effect = 1+(100-1)*(ICC=.01)=2*
