# Supplementary material for: Impact of Maternal HIV Seroconversion during Pregnancy on Early Mother to Child Transmission of HIV (MTCT) Measured at 4-8 Weeks Postpartum in South Africa 2011-2012: A National Population-Based Evaluation
Source: PLoS One. 2015 May 5;10(5):e0125525. doi: 10.1371/journal.pone.0125525 (PMC4420458; doi:10.1371/journal.pone.0125525)
Supplement: S2 Table — (DOCX) [file pone.0125525.s002.docx]

**Table S2. Distribution of individual variables that contributed to the overall socio-economic status (SES) score levels of average, lower than average and lowest SES**.

SES variable was a composite variable of 10 different variables constructed using a clustering algorithm (Spath, H. (1980), *Cluster Analysis Algorithms*, Chichester, Eng.: Ellis Horwood.; Hartigan, J. A. (1985), "Statistical Theory in Clustering," *Journal of Classification*, 2, 63–76. SAS STAT 9.2 Documentation.) that considered 10 interview items that measured a spectrum of socio-economic indicators, and used the distance between an observations value on each of these variables and the overall mean for that variable to create three SES levels for the population. Table S2 shows the distribution of these variables across the 3 levels of our calculated SES variable for the entire study sample. The largest differences between the lower and lowest SES groups were the availability of electricity or gas for cooking, and access to home amenities such as a stove, radio, television or telephone. There was not a significant difference between the lower and lowest SES groups in terms of reported food scarcity, however, women in the lowest SES group were less likely to receive support from a male partner and more likely to receive a Child support grant from the South African government. (<http://www.services.gov.za/services/content/Home/ServicesForPeople/Socialbenefits/childsupportgrant/en_ZA> ; <http://www.info.gov.za/view/DownloadFileAction?id=90553>

| **Variable included in the SES factor analysis** |  | **Average SES %**  **(95% Confidence Interval)** | **Lower SES %**  **(95% Confidence Interval)** | **Lowest SES %**  **(95% Confidence Interval)** |
| --- | --- | --- | --- | --- |
| Home Material | Brick | 90.8 (89.5-92.2) | 49.4 (45.8-52.9) | 42.3 (34.6- 49.9) |
|  | Informal material/corrugated iron/wood | 9.2 (7.8-10.5) | 29.3 (25.6-32.9) | 27.5 (22.1-32.9) |
|  | Traditional/Mud | 0.0 | 21.3 (17.5-25.3) | 30.2 (19.4-41.0) |
| Water Source (Piped vs. not piped) | Piped | 91.8 (90.5-93.0) | 41.0 (36.5-45.5) | 32.5 (27.6-37.4) |
| Toilet Type | Flush | 77.7 (75.5-79.8) | **1.9 (1.3-2.5)** | **0** |
|  | Pit Latrine | 22.3 (20.2-24.5) | **97.6 (96.9-98.2)** | **53.6 (43.8-63.4)** |
|  | None/Other | 0.0 | 0.5 (0.2-0.8) | 46.4(36.6-56.2) |
| Cooking Fuel | Electricity/Gas | 99.3 (99.1-99.5) | **79.0 (75.6-82.4)** | **39.5 (31.5-47.7)** |
|  | Wood/coal | 0.7(0.4-0.9) | **21.0(17.5-24.4)** | **60.4 (52.3-68.5)** |
| Household owns | Refrigerator | 87.8 (86.4-89.1) | **56.0(53.1-58.9)** | **12.7 (9.7-15.7)** |
|  | Radio | 82.7(81.3-84.2) | **73.7 (71.4-76.2)** | **36.4 (30.1-42.7)** |
|  | Television | 91.3(90.3-92.3) | **70.4(68.1-72.7)** | **16.0(12.9-19.1)** |
|  | Stove | 97.3 (96.7-97.9) | **84.7(82.1-87.2)** | **41.7(36.4-47.0)** |
|  | Landline Telephone / Cell phone | 92.7(91.9-93.6) | **90.5(89.0-92.1)** | **73.3(69.3-77.3)** |
|  | Car | 17.8(16.5-19.2) | 5.7 (4.7-6.8) | 3.4(1.7-5.0) |
| Variables expected to be associated with socio-economic status used to test the face validity of the clustering procedure. | | | | |
| In the last year was there a time when the family ran out of food and had to ask for help? (Yes) | | 9.8 (8.6-11.0) | 14.6(12.4-16.7) | 33.7(28.8-38.5) |
| Source of maternal income | |  |  |  |
|  | Mother’s employment | **21.4 (20.1-22.8)** | **10.4(9.0-11.7)** | **6.8 (5.0-8.7)** |
|  | Partner/Husband/Ex-husband | **65.9 (64.1-67.8)** | **57.4 (54.7-60.0)** | **44.4 (39.8-49.1)** |
|  | Child Support grant | **11.1(9.8-12.2)** | **20.5 (18.3-22.7)** | **33.9 (27.9-39.9)** |
