# Supplementary material for: Impact of Maternal HIV Seroconversion during Pregnancy on Early Mother to Child Transmission of HIV (MTCT) Measured at 4-8 Weeks Postpartum in South Africa 2011-2012: A National Population-Based Evaluation
Source: PLoS One. 2015 May 5;10(5):e0125525. doi: 10.1371/journal.pone.0125525 (PMC4420458; doi:10.1371/journal.pone.0125525)
Supplement: S3 Table — (DOCX) [file pone.0125525.s003.docx]

**Table S3: Weighted risk factors of the maternal HIV seroconversion during pregnancy, South Africa, 2011-2012 (Full model)**

|  |  | *Adjusted* Odds Ratio Estimates* | | | *Adjusted* Hazard Ratio Estimates* | | |
| --- | --- | --- | --- | --- | --- | --- | --- |
| *Effect* | *Weighted row % of HIV seroconversion (3.29%)* | *Point Estimate* | *95% Wald Confidence Limits* | | *Point Estimate* | *95% Wald Confidence Limits* | |
| Each additional year in mother’s age |  | 1.06 | 1.03 | 1.09 | 1.06 | 1.03 | 1.09 |
| Each additional ANC visit (over 1) |  | 0.90 | 0.83 | 0.97 | 0.89 | 0.82 | 0.97 |
| Maternal education completed |  |  |  |  |  |  |  |
| No Education | 2.83 | 1.81 | 0.50 | 6.53 | 1.83 | 0.52 | 6.46 |
| Grade 1-7 | 5.09 | 2.67 | 1.04 | 6.85 | 2.64 | 1.04 | 6.67 |
| Grade 8-12 | 3.18 | 1.91 | 0.81 | 4.53 | 1.91 | 0.81 | 4.46 |
| >12 years | 1.57 | Ref. |  |  | Ref. |  |  |
| Baby’s father HIV status |  |  |  |  |  |  |  |
| HIV-negative | 2.18 | Ref. |  |  | Ref. |  |  |
| HIV-infected | 10.74 | 5.09 | 1.23 | 21.02 | 4.83 | 1.28 | 18.15 |
| Don’t know | 4.31 | 1.94 | 1.42 | 2.65 | 1.93 | 1.42 | 2.61 |
| Tuberculosis screening during pregnancy |  |  |  |  |  |  |  |
| Yes | 4.64 | 1.76 | 1.35 | 2.30 | 1.74 | 1.34 | 2.26 |
| No |  | Ref |  |  | Ref. |  |  |
| Birth Attendant |  |  |  |  |  |  |  |
| Traditional Healer | 6.69 | Ref. |  |  | Ref. |  |  |
| Nurse/Midwife | 3.33 | 0.49 | 0.23 | 1.07 | 0.49 | 0.24 | 1.04 |
| Doctor | 2.70 | 0.43 | 0.18 | 1.00 | 0.43 | 0.19 | 0.98 |

*Factors associated with HIV seroconversion during pregnancy were assessed using proportional hazard models using a multiple imputation dataset. Estimates are adjusted for clustering, nonresponse, and weighted by SA’s 2011 live-births (see the method of the main manuscript for more detail). *Adjusted for all listed covariates, total pregnancies including the index pregnancy, and the amount of support the mother reported receiving during pregnancy weighted to account for sampling design and non-response, and with variance estimation via Taylor series linearization to account for sampling design (see methods).*

*We controlled for all listed covariates as well as mother’s marital status, socio-economic status, number of live-children and parity including the index pregnancy, maternal knowledge of modes of HIV transmission and receipt of support during pregnancy), and whether the infant was born at home, in a hospital or clinic.*
